# Adaptive constraints by morphological operations for single-shot digital holography

**DOI:** 10.1038/s41598-023-37423-3

**Published:** 2023-06-24

**Authors:** Danlin Xu, Zhengzhong Huang, Liangcai Cao

**Affiliations:** 1 State Key Laboratory of Precision Measurement Technology and Instruments, Department of Precision Instruments, Tsinghua University, Beijing, 100084 China; 2 School of Information and Optoelectronic Science and Engineering, South China Normal University, Guangzhou, 510006 China

**Keywords:** Imaging and sensing, Optical sensors

## Abstract

Digital holography provides access to quantitative measurement of the entire complex field, which is indispensable for the investigation of wave-matter interactions. The emerging iterative phase retrieval approach enables to solve the inverse imaging problem only from the given intensity measurements and physical constraints. However, enforcing imprecise constraints limits the reconstruction accuracy and convergence speed. Here, we propose an advanced iterative phase retrieval framework for single-shot in-line digital holography that incorporates adaptive constraints, which achieves optimized convergence behavior, high-fidelity and twin-image-free reconstruction. In conjunction with morphological operations which can extract the object structure while eliminating the irrelevant part such as artifacts and noise, adaptive constraints allow the support region to be accurately estimated and automatically updated at each iteration. Numerical reconstruction of complex-valued objects and the capability of noise immunity are investigated. The improved reconstruction performance of this approach is experimentally validated. Such flexible and versatile framework has promising applications in biomedicine, X-ray coherent diffractive imaging and wavefront sensing.

## Introduction

Phase contains fundamental information about the optical properties and characteristics of objects. However, the oscillation frequencies of light waves are so fast that the imaging sensors enable to measure only the intensity of the field while the phase is missing^1^. Retrieval of the phase becomes a challenging issue. Digital holography is a label-free and non-invasive imaging technique to extract the phase, referring to digital record of holograms and numerical reconstruction by means of diffraction theory^2^. Holograms captured by an electronic sensor encode both the amplitude and the phase of the object’s wavefront through introducing a reference light. Digital holography provides some significant advantages, including high-speed holographic wavefront acquisition, availability of multi-dimensional information without requiring an imaging lens and versatility of image processing techniques^3,4^, which promises practical applications in microscopy^5,6^, biological specimen analysis^7–9^, terahertz waves^10,11^ and microfluidics^12^.

Based on Gabor’s holography, the in-line digital holography has emerged as an attractive and simple holographic configuration, where the axes of the diffracted object wave and the reference wave are parallel^13^. Although the in-line setup endows with full bandwidth utilization and high phase sensitivity, the quality of reconstructed images is susceptible to the overlapping out-of-focus twin-image artifact that exists owing to the on-axis incidence of the two beams^14^. As a result, varieties of experimental means have been implemented to address the twin-image noise^15–19^, such as off-axis holography^15^, phase-shifting^16,17^ and so on. Nevertheless, the off-axis holography requires an oblique-angle reference beam, resulting in the sacrifice of space-bandwidth product of the imaging system. In terms of the phase-shifting strategy, a time-division phase-shifting method needs sequential exposure of phase-shifted holograms at the expense of time-bandwidth product, whereas a wave-splitting phase-shifting method requires specially designed optical elements^20^. More recently, numerical approaches have been explored and developed to tackle with the twin-image problem^21–23^, wherein phase retrieval algorithm is typically employed.

Phase retrieval aims to recover a complex-valued signal given intensity-only diffraction patterns, which is supposed to be a key ingredient of in-line digital holography^24^. As a wavefront-sensing method, phase retrieval has attracted widespread attention for the reason that it offers a solution to the phase problem arising in diverse fields including crystallography^25^, astronomy^26^ and optical imaging^27^. In order to generate better behaved image reconstruction models to meet practical applications, different techniques have been introduced to optimize phase retrieval problem, such as deep learning^28–30^, modified sensor masks^31^ and pixel super resolution^5,14^. The pioneering research work of iterative phase retrieval dates back to Gerchberg–Saxton (GS) algorithms which is slow and sensitive to initial guesses^32^, therefore inspiring a furry of follow-up work to improve the GS iterative framework^33–35^. Such GS-based algorithms exploit back-and-forth propagation between different planes, embedding physical constraints like support^36–38^, non-negativity^39,40^, absorption^21^ into iteration to speed up convergence and avoid local convergence during iteration. Support constraints require the prior knowledge of the object shape, so as to set the transmission function of the object beyond the known object boundary to zero. To escape the need of such prior knowledge, the adaptive support of “shrink-wrap” technique based in the spatial^37^ and frequency^38^ domain estimates and updates the support area according to the range where the intensity is over a certain threshold. However, if the determined threshold level is lower, the support region could be overestimated which induces the residue of noise in the reconstruction. While if the determined threshold level is higher, some object information could be missing. The inaccurate selection of the threshold causes a less than perfect estimation of the support. Thus, the absorption constraint has been proposed relying on the principle that absorption may not give rise to an increased amplitude following a scattering process. Although such effective method dispenses with the object support, it is limited to the samples which do not absorb or scatter optical wave significantly^41^. Recent researches favor multi-image phase retrieval because it is flexible to the issues existing in the GS-based algorithms. Multiple frames of raw images serve as amplitude constraints to force the complex object field to gradually agree well with all these measurements. The data redundancy supplied by multiple measurements is capable of reconstructing complex-valued objects^42^. Multiple measured holograms can be achieved by varying object-to-sensor distances^38,43–45^, illumination wavelengths^46,47^, illumination angles^48,49^ and coding mask modulation^50^. Unfortunately, the achievable of a stable and accurate reconstruction is strongly enslaved to high-precision controllable devices, slow convergence rate, great quantities of holographic data and iterations. Hence, optimizing the phase retrieval approach for high-quality reconstruction and improved noise immunity still remains a challenging task.

Morphological filtering technique is a nonlinear signal processing method stemming from set theory and integral geometry^51^, which is developed to numerous applications such as biomedical image processing, machined surface inspection and fault diagnosis. Typically, morphological operations, comprising erosion, dilation, closing, opening, related combined and compound operations, can be employed to modify the geometry of the raw image, thereby to realize the extraction of the exact pictorial information. In this work, we propose an advanced iterative phase retrieval method that generates adaptive constraints by morphological operators for single-shot in-line digital holography to improve the reconstruction quality and speed up convergence. In this proposed scheme, the square root of a single intensity pattern is used to update the modulus of the diffracted wave field in the diffraction space, and the adaptive constraint updated iteratively by morphological operators is applied to confine the object wave field, so that it can adaptively tend to the accurate amplitude and phase distributions of a complex object and accelerate the convergence speed. Compared to previous strategies for generating the object support, the proposed approach in conjunction with morphological operations provides a more appropriate and efficient estimation of support. The support region generated automatically is tight enough to eliminate the need of a sequence of intensity patterns. The method of generating adaptive constraints by morphological operations is described in detail. The numerical calculation and the immunity to noise are discussed. Moreover, the experimental reconstructions are presented to verify the stability and accuracy of our approach.

## Methods

### Optical setup for in-line digital holographic imaging

**Figure 1:**
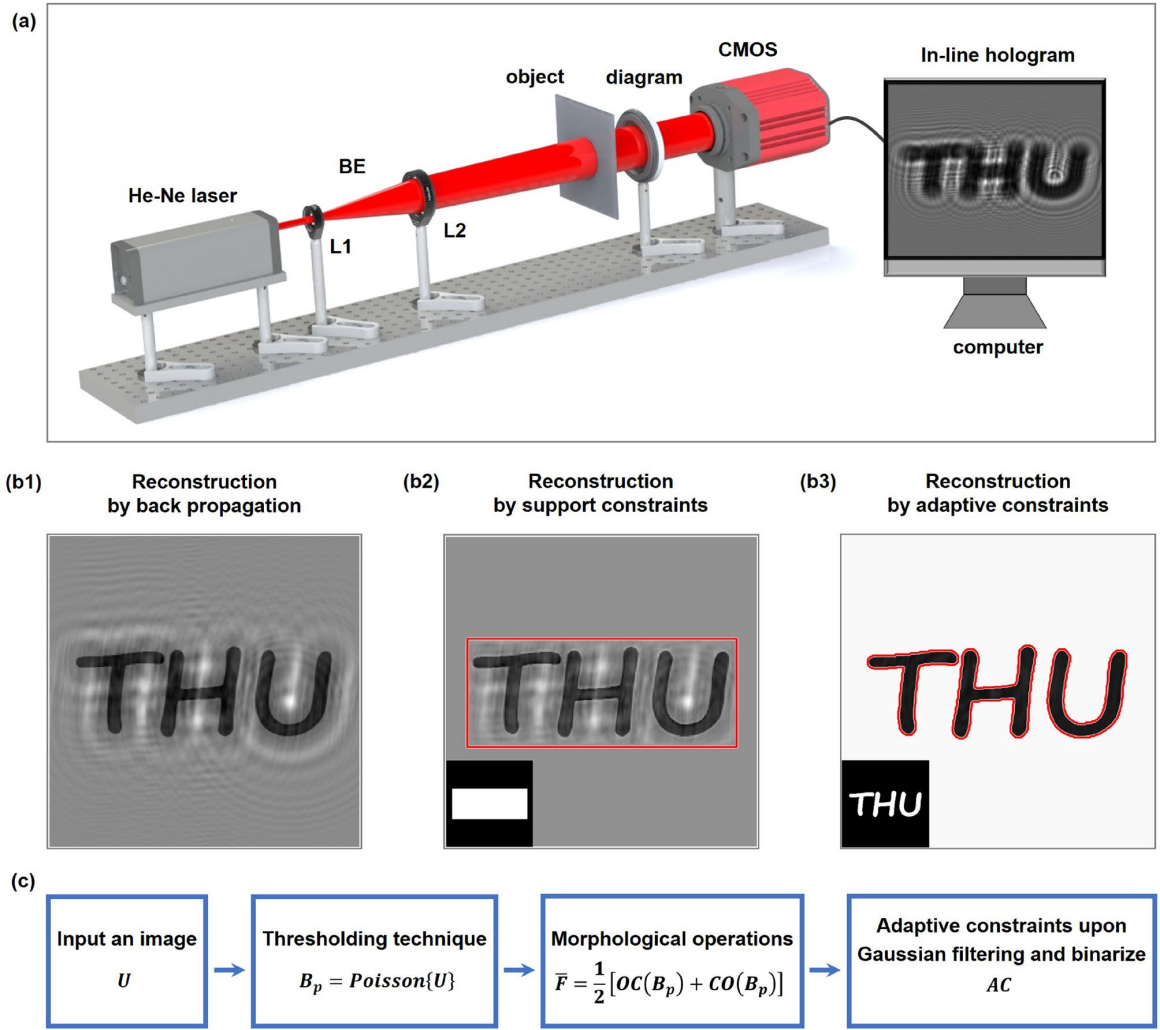
(**a**) Optical configuration of an in-line holographic imaging system. BE, beam expander; L1–L2, lenses. (**b1**) In-line reconstruction by back-propagating to the object plane. (**b2**) Reconstruction by applying support constraints in the iterative phase retrieval method after 40 iterations. (**b3**) Reconstruction by applying adaptive constraints in the iterative phase retrieval method after 40 iterations. The support region is outlined by red lines and the constraint patterns are situated in the lower left corner respectively. (**c**) Schematic diagram for the steps of generating adaptive constraints.

Here, an in-line digital holographic imaging configuration is considered, which is presented in Fig. 1a. A linearly polarized Gaussian laser beam is emitted from a He–Ne laser at the wavelength of 632.8 nm. After being collimated and expanded by the beam expander (BE) which is comprised of two lenses, the laser beam passes through the object. Then the interference of the scattered and unscattered wave fields generates an in-line hologram recorded by a CMOS sensor (QHY174, QHYCCD Co., Ltd.). The CMOS sensor we used has the resolution of $1200 \times 1920$ with a pixel size of 5.86 $\upmu$m, and the field of view (FOV) of such holographic imaging system is $7.032 \times 11.251$ mm$^2$. On account of the limited FOV of the CMOS, the diagram acts as a low-pass filter to select a certain area of the object to image on the CMOS. Such lensless in-line digital holographic imaging system is characterized by portability, low cost and high space-bandwidth product.

### Principles of adaptive constraints applied in iterative phase retrieval

Mathematically, the phase-retrieval problem can be formulized as^52^

1 $$\begin{aligned} Find \ {\varvec{u}} \in {\mathbb {C}} \ \ \ \ \ \ \ \ s.t. \ \ \ {{\varvec{I}}_k} = {\left| {{{{\varvec{A}}}_k}\left( {{{{{\textbf {M}}}}_k} \odot {\varvec{u}}} \right) } \right| ^2}, k = 1,\ldots ,N, \end{aligned}$$

where ${{\textbf {u}}} \in {\mathbb {C}}$ is the complex-valued signal representing the transmission of the interested object, ${{\textbf {I}}}$ is the measured intensity pattern, ${\varvec{A}}$ expresses the wavefront propagation process, $\odot$ refers to the Hadamard product, ${\varvec{M}}$ represents the modulated optical masks that provides constraints to optimize the convergence and reduce the ill-posedness of the inverse problem. Early researches concentrated on introducing support constraints which limit the reconstructed region into the double-side constraint iteration. Specially, the mathematical definition of the support constraint describes as $${u_i} = \left\{ {\begin{array}{*{20}{c}} {{u_i}}&{}{i \in S}\\ 0&{}{i \notin S} \end{array}} \right.$$, where *S* denotes the set of pixels within the support region. The conventional backpropagation reconstruction overlapped with twin image is presented in Fig. 1b1. In the iterative phase retrieval method, the support constraint is commonly imposed on the object domain. Given the prior knowledge of the object size, the support constraint is generally assumed as a rectangular or circular aperture to eliminate the information located outside the estimated support region^53^. As seen in Fig. 1b2, such loose and rough support enables to suppress artifacts outside the boundaries, but artifacts distributed within the support region still preserve, which significantly obfuscates the reconstruction. Moreover, in terms of the target object in a particular shape, designing a specific support is complicated and inefficient. Hence, morphological operations are introduced to generate adaptive constraints, aiming to improve the accuracy and efficiency of extracting the relevant image structures and solve the twin-image problem.

Morphological filtering, mainly involving morphological operations and structural element (SE), is regarded as a powerful image processing tool for analyzing and extracting the geometrical structure of an image even wrapped by noise. The interactions between a raw image and a certain pre-designed SE can be investigated by morphological operations^51^. A binary SE represents a smaller matrix of pixels than that of the raw image, whose features are determined by its shape and domain. Mathematically, the binary erosion of a set *F* by a pre-defined SE (*G*) is expressed as^54^

2 $$\begin{aligned} F\Theta G = \left\{ {\varepsilon |{{\left( G \right) }_\varepsilon } \subseteq F} \right\} , \end{aligned}$$

where $\Theta$ represents the erosion operation, ${\left( G \right) _\varepsilon } = \left\{ {\sigma |\sigma = g + \varepsilon ,g \in G} \right\}$ denotes the translation of origin of *G* to point $\varepsilon$. It means that erosion replaces each pixel with the local minimum of all pixels in the neighborhood whose shape and size is determined by SE. On the contrary, the binary dilation replaces each pixel with the local maximum of all pixels in the neighborhood, which is defined as^54^

3 $$\begin{aligned} F \oplus G = \left\{ {\varepsilon |{{\left( {{\hat{G}}} \right) }_\varepsilon } \cap F \ne \emptyset } \right\} , \end{aligned}$$

where $\oplus$ represents the dilation operation, ${\hat{G}}$ is the reflection of *G*. Based on the combination of erode and dilation operations, the opening and closing operations can be described respectively as

4 $$\begin{aligned} \begin{aligned} F \circ G = \left( {F\Theta G} \right) \oplus G, \\ F \bullet G = \left( {F \oplus G} \right) \Theta G, \end{aligned} \end{aligned}$$

where $\circ$ denotes the opening operation and $\bullet$ denotes the closing operation. Morphological opening operation eliminates the irrelevant part such as scatters and burrs in the image while preserving the primary geometry of the object structure. Morphological closing operation fills the small holes and thickens the foreground pixels in the image. Because the morphological opening and closing functions are not mutually inverse^55^, they can be further cascaded as open-closing and close-opening operations by using different SE (*G*1, *G*2), which can be considered as

5 $$\begin{aligned} \begin{aligned} {OC(F) = \left( {F \circ {G_1}} \right) \bullet {G_2},}\\ {CO(F)} = \left( {F \bullet {G_1}} \right) \circ {G_2}. \end{aligned} \end{aligned}$$

On the basis of the skeletons and morphological characteristics in an image, morphological operations have the ability to selectively remove the unwanted structures such as noise and unrelated target objects. The reconstruction shown in Fig. 1b3 indicates that using adaptive constraints obviously enhances extraction capability and removes disturbance of twin-image artifacts. It offers a more appropriate and efficient estimation of object support. Stages for the generation of adaptive constraints are illustrated in Fig. 1c. An input image *U* is firstly binarized using Poisson distribution-based threshold^56^. Such automatic threshold algorithm dispenses with the need for setting a certain threshold and optimizing any parameters. Then the morphological adjustments are considered for refining the image. Due to the shrinkage of the closing operator and the expansibility of the opening operator^55^, close-opening filter enlarging the object domain incurs the inadequate removal of noise, while open-closing filter contracting the object domain causes the partial loss of relevant image information. To get the trade-off between these two filters, an average weighted combination of open-closing and close-opening operations ${\bar{F}} = \frac{{{OC}\left( {{B_p}} \right) + {CO}\left( {{B_p}} \right) }}{2}$ is calculated. After undergoing morphological operations, Gaussian filtering is applied to smooth the image ${\bar{F}}$. Finally, the adaptive constraint is formed upon binarizing the filtering image again according to the threshold obtained by edge detection method ‘Sobel’. If the constraint is created by using the threshold-based segmentation algorithm in a straightforward manner, the binary pattern depends on the area where the intensity is above the pre-assessed threshold. In this case, the accuracy of estimating the object support is susceptible to twin-image artifacts and measurement noise in experiment. Instead, morphological filtering enables to extract the geometrical structure of featured objects even in cases involving experimental noise, utilizing a SE to probe each pixel and modify its grayscale value according to the intensity of all pixels in its neighborhood. Consequently, the adaptive constraint generated by morphological filtering is updated at each iteration, giving rise to a sharper and tighter support region.

**Figure 2:**
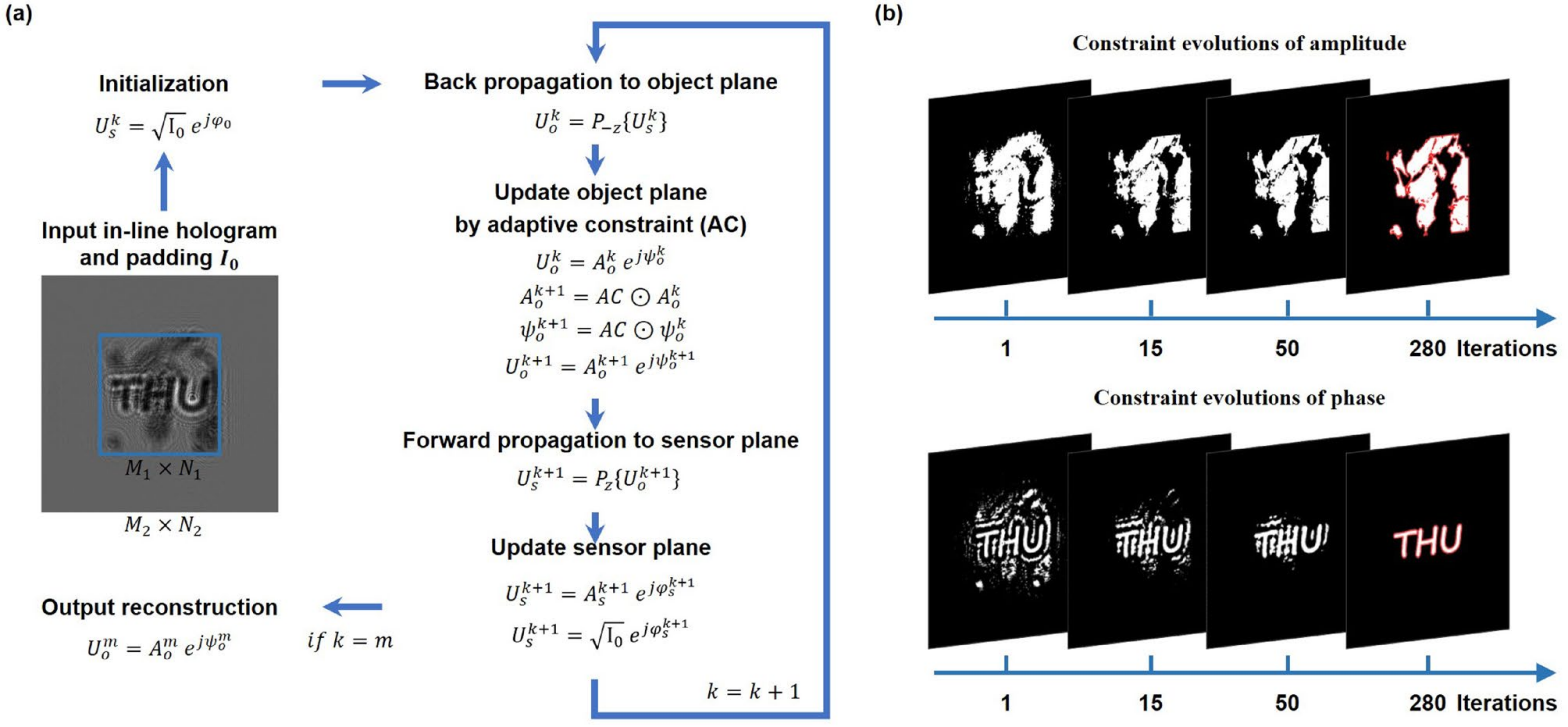
(**a**) The overview flowchart of adaptive constraints inserted in the iterative phase retrieval of a complex-valued object. (**b**) Evolutions of adaptive constraints versus iterations which is applied to adaptively update the amplitude and phase distributions in the object plane.

In pursuit of an advanced iterative phase retrieval method for in-line hologram that endows with high-fidelity reconstruction, superior convergence speed and improved immunity to noise, adaptive constraints by morphological operations are introduced to the iterative phase retrieval algorithm. The overview flowchart is depicted in Fig. 2a. The iterative process between the sensor plane and the object plane is performed as follows.

- Step 1: Initialization. The in-line hologram with ${M_1} \times {N_1}$ pixels captured by the CMOS is padded with constant to $I_0$ with ${M_2} \times {N_2}$ pixels (${M_2}> {M_1}, {N_2} > {N_1}$). The initial amplitude is identified as the square root of the padding hologram, and the initial phase ${\varphi _0}$ is set to random or constant. Subsequently, the initial complex-valued wave field is expressed as $U_s^k = \sqrt{{I_0}} \exp \left( {j{\varphi _0}} \right) .$
- Step 2: The process of the wave field propagating in the free space is calculated by the angular spectrum method (ASM)^57^, which takes the form as
  6 $$\begin{aligned} {P_z}\left( u \right) = {{\mathscr {F}}^{ - 1}}\left\{ {{\mathscr {F}}\left\{ u \right\} \cdot H\left( {{f_x},{f_y},z} \right) } \right\} , \end{aligned}$$
  where ${\mathscr {F}}$ and ${\mathscr {F}}^{ - 1}$ represent two-dimensional Fourier transform and inverse Fourier transform respectively, *H* is the transfer function defined as
  7 $$\begin{aligned} H\left( {{f_x},{f_y},z} \right) = \left\{ {\begin{array}{*{20}{c}} {\exp \left( {i2\pi z\sqrt{\frac{1}{{{\lambda ^2}}} - f_x^2 - f_y^2} } \right) ,}&{}{f_x^2 + f_y^2 \le \frac{1}{{{\lambda ^2}}}}\\ 0 &{}{otherwise} \end{array}} \right. , \end{aligned}$$
  where $({f_x},{f_y})$ is the coordinate in spatial frequency domain, z denotes the distance from the object plane to the sensor plane. In digital holography, there are various types of reconstruction noise that can affect the quality and accuracy of the reconstruction. In order to achieve a better reconstruction, it is important to acquire the focus distance of an object. The optimal reconstruction plane can be identified based on Tamura coefficient (TC) metric which has only a single extreme in the whole focus range^58^. Hence, taking advantage of the intrinsic flexibility of digital holography due to its numerical focusing ability, multiple sections of an object can be reconstructed by locating the best focus distance corresponding to different sections of the object. $\lambda$ is the wavelength. The complex-amplitude field $U_s^k$ distributed in the sensor plane is propagated back to the object plane to obtain $U_o^k = {P_{-z}}\left( {U_s^k} \right) = A_o^k\exp \left( {j \psi _o^k} \right)$.
- Step 3: In the object plane, the adaptive constraint (*AC*) is implemented to update both the amplitude $A_o^k$ and the phase $\psi _o^k$. The generation of adaptive constraints combines thresholding technique and morphological operations, which is illustrated in Fig. 1c in detail.
- Step 4: By propagating the updated field $U_o^{k+1}$ forward to the sensor plane, the complex-amplitude field is given by $U_s^{k+1}={P_z}\left( {U_o^{k+1}} \right)$. Then, the amplitude of the complex-valued field in the sensor plane $U_s^{k+1}=A_s^{k+1}\exp \left( {j \varphi _s^{k+1}} \right)$ is updated with square root of the in-line hologram, while the phase value is still maintained.

Steps 2–4 are processed repeatedly until the $m_{th}$ iteration. Finally the reconstruction can be achieved by back propagating from the sensor plane to the object plane. The free-space propagation is calculated as a circular convolution model via fast Fourier transforms(FFTs), which causes wraparounds superimposed on the calculation result. In order to resolve this issue, adequate padding is used to prevent periodization artifacts and obtain an accurate calculation^59^. Evolutions of adaptive constrains enforced on the amplitude and the phase in the object plane are exhibited in Fig. 2b. The adaptive constraints are generated at each iteration by employing morphological operations based on the reconstructed amplitude and phase in the object plane respectively. The implementation of morphological filtering helps to extract the object structure while simultaneously eliminating the artifact and noise. As the iteration process continues, the constraint is updated automatically and performs a Hadamard product with the reconstructed images in the object plane. Hence, irrelevant regions are gradually filtered out, causing the support region to automatically shrink to match the geometrical structure of the object. The generation of adequate tight and sharp support region is conducive to promote the accuracy of extracting the object information and suppress the overlapping twin-image artifact that obscures the reconstruction.

## Numerical calculation

### Reconstruction of complex-valued objects

For the purpose of verifying the improvement effect of employing adaptive constraints in the iterative phase retrieval, the comparison of reconstruction quality and convergence behavior with multi-distance phase retrieval (MPR), support and adaptive constraints is carried out. The parameters we used for numerical simulation are as follows: the wavelength is 500 nm, the pixel pitch is 5.86 $\upmu$m, *G*1 creates a disk-shaped SE with a radius equal to 1 and *G*2 creates a $2 \times 2$ square SE. In-line holograms with $500 \times 500$ pixels are padded with constant to an image with $1000 \times 1000$ pixels. Given an in-line hologram with the imaging distance of 6cm, support and adaptive constraints are enforced on the object plane in the double-side constraint iteration. For the MPR method, We adopted three in-line holograms with a distance interval of 0.5 mm. These three intensity patterns are treated as amplitude constraints in the iterative phase retrieval.

**Figure 3:**
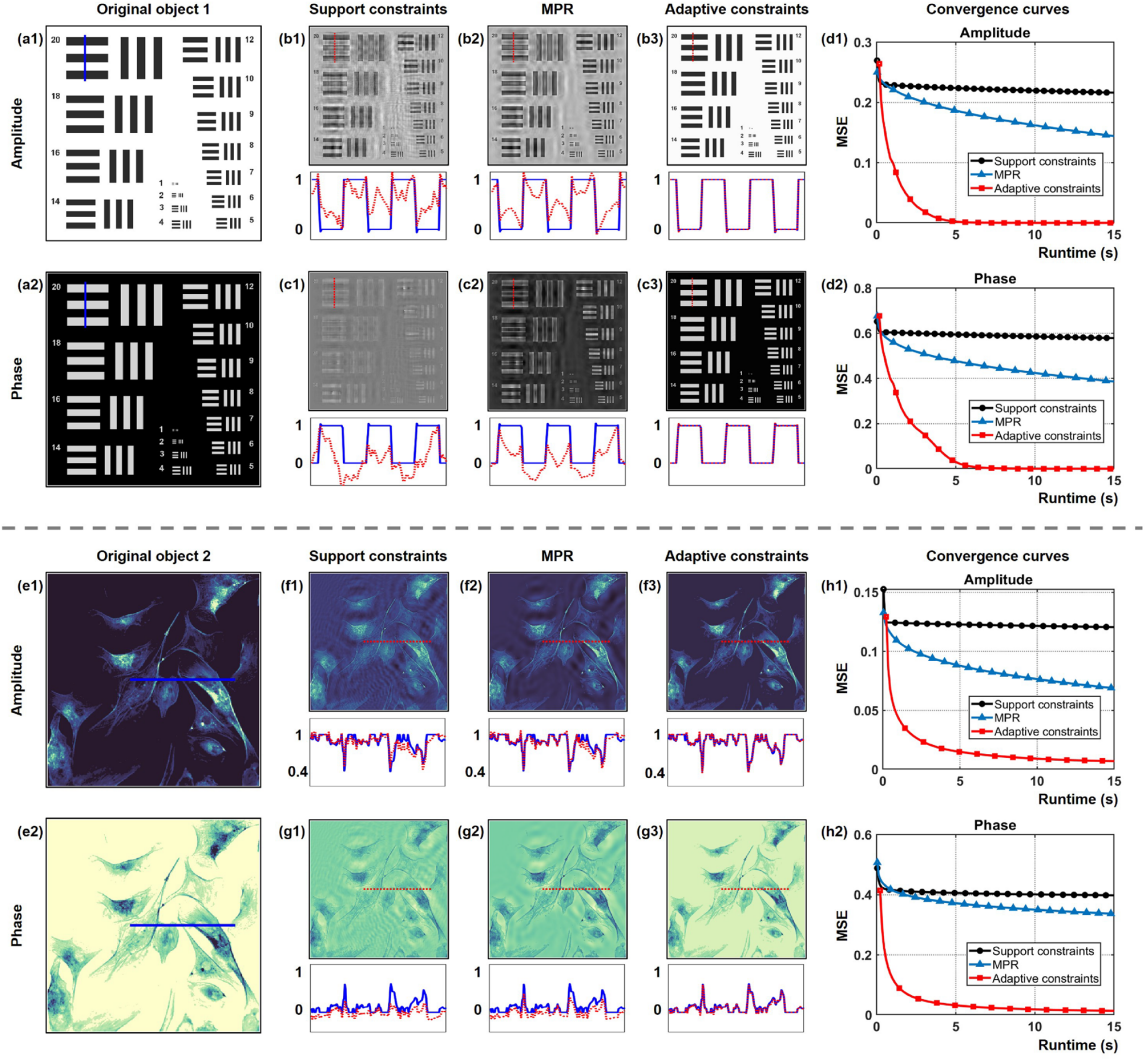
Reconstruction of a complex-valued object with “flat” boundary by using support constraints, MPR and adaptive constraints. (**a1**,**a2**) Ground-truth amplitude and phase of the original object 1. (**b1**–**b3**) Comparison of the retrieved amplitude after 200 iterations. (**c1**–**c3**) Comparison of the retrieved phase after 200 iterations. (**d1**–**d2**) The MSE curves against runtime of the retrieved amplitude and phase in object 1. (**e1**,**e2**) Ground-truth amplitude and phase of the original object 2. (**f1**–**f3**) Comparison of the retrieved amplitude after 100 iterations. (**g1**–**g3**) Comparison of the retrieved phase after 100 iterations. (**h1**,**h2**) The MSE curves against runtime of the retrieved amplitude and phase in object 2. Below are the cross-sectional profiles, where the red line indicates the retrieved amplitude and phase value, and the blue line indicates the amplitude and phase value of the original object.

In Fig. 3a1,a2, the amplitude and phase distributions of a complex-valued object with “flat” boundary serve as ground truth. The retrieved amplitude and phase by methods of support constraints, MPR and adaptive constraints are shown in Fig. 3b1–c3. Note that the reconstructions by support constraints and MPR still suffer from the overlapping twin-image artifact. That is because the support constraint seems as an aperture that mainly contributes to filtering artifacts beyond the object support region, but artifacts wrapped inside the pre-designed boundary can not be obstructed during iterations. MPR approach is able to achieve stable and high-fidelity reconstruction, but the reconstruction quality is restricted by the quantity of captured holograms and iterations. In contrast with them, incorporating adaptive constrains into the iterative phase retrieval exhibits higher-quality reconstruction and better ability to tackle with the twin-image problem. The twin-image artifact is interpreted as noise terms that can be removed by morphological operations. The cross-sectional profiles match well with ground truth. In addition to reconstructing the object with stepped-phase distributions, comparison of reconstructing the object with continuous-phase distributions by means of different phase retrieval algorithms is also discussed. According to ground truth of object 2 shown in Fig. 3e1, e2, it is noticeable that the retrieved amplitude and phase using adaptive constraints still have a better performance in reconstruction quality and twin-image elimination than that using support constraints and MPR, which is depicted in Fig. 3f1–g3. Applying conventional support constraints generally suffers from a high reconstruction error because the loose support region is insufficient to suppress the artifact. By introducing the morphological filtering, adaptive constraints allow for gradually adjusting the support domain according to the structural characteristics of the object during iterations. The mean square errors (MSEs) $E = \left[ {\sum \nolimits _{x,y} {{{\left| {\rho \left( {x,y} \right) - {\rho _0}\left( {x,y} \right) } \right| }^2}} } \right] /\left[ {\sum \nolimits _{x,y} {{{\left| {{\rho _0}\left( {x,y} \right) } \right| }^2}} } \right]$ of the retrieved amplitude and phase considered as a metric to reflect the reconstruction quality with different approaches are calculated in Fig. 3d1,d2,h1,h2, where $\rho \left( {x,y} \right)$ is the estimated distribution and ${\rho _0}\left( {x,y} \right)$ is the initial distribution. It is indicated that the method of incorporating adaptive constraints is allowed to achieve a more optimized convergence performance.

**Figure 4:**
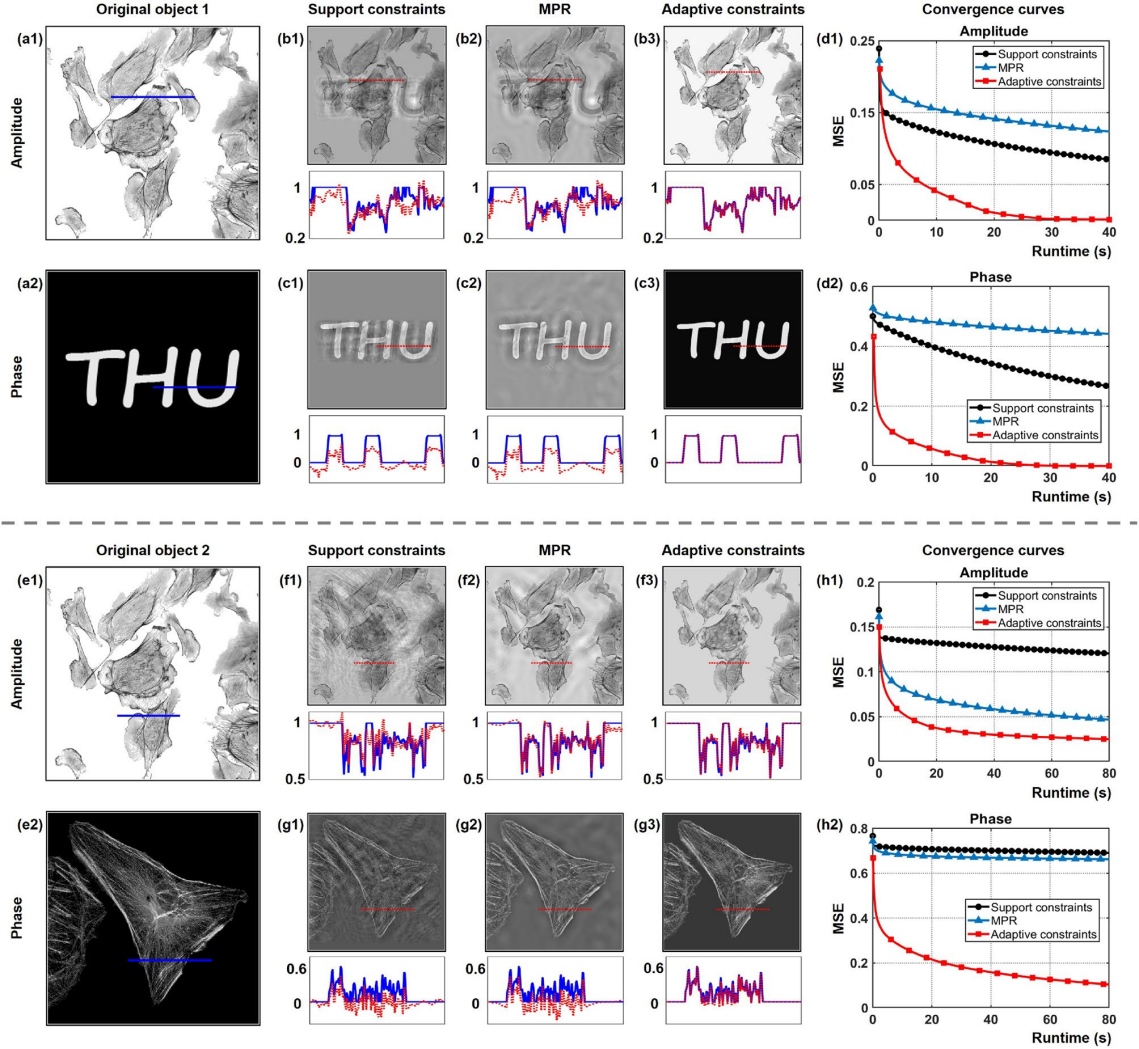
Reconstruction of a complex-valued object with “random” boundary by using support constraints, MPR and adaptive constraints. (**a1**,**a2**) Ground-truth amplitude and phase of the original object 1. (**b1**–**b3**) Comparison of the retrieved amplitude after 300 iterations. (**c1**–**c3**) Comparison of the retrieved phase after 300 iterations. (**d1**,**d2**) The MSE curves against runtime of the retrieved amplitude and phase in object 1. (**e1**,**e2**) Ground-truth amplitude and phase of the original object 2. (**f1**–**f3**) Comparison of the retrieved amplitude after 600 iterations. (**g1**–**g3**) Comparison of the retrieved phase after 600 iterations. (**h1**,**h2**) The MSE curves against runtime of the retrieved amplitude and phase in object 2. Below are the cross-sectional profiles, where the red line indicates the retrieved amplitude and phase value, and the blue line indicates the amplitude and phase value of the original object.

Another example of reconstructing a complex-valued scene with “random” boundary is depicted in Fig. 4. Figure 4a1,a2 indicate the ground-truth amplitude and phase of the original object 1. A image with sharp edges is input as the phase part. As shown in Fig. 4b1–c3, the reconstruction quality by support constraints and MPR methods is severely degraded by artifacts originating from mutual interference between the amplitude and the phase distributions of the object with “random” boundary. Significantly, artifacts can be completely removed by imposing adaptive constraints, which demonstrates that adaptive constraints generated by morphological operations help to accurately extract the object structure and simultaneously eliminate the unwanted information. To further visualize the reconstruction effect of applying adaptive constraints, a cell image with more complicated distribution is used as the phase part shown in Fig. 4e2. In Fig. 4f1–g3, it is observed that the use of support constraints is ineffective for retrieving the low-frequency phase information, and the reconstruction is obscured by the artifact. Enforcing adaptive constraints on the object plane appears to be a viable solution for these issues, as it enables the rendering of recognizable cellular structures. It can also be discovered in Fig. 4d1,d2,h1,h2 that adaptive constraints require significantly less time to achieve the same MSE as the other phase retrieval approaches, which illustrates that employing adaptive constraints has higher convergence speed and lower MSE. Therefore, adaptive constraints incorporated into the phase iteration method have improvement in fidelity of reconstruction and convergence behavior.

### Reconstruction under noisy conditions

**Figure 5:**
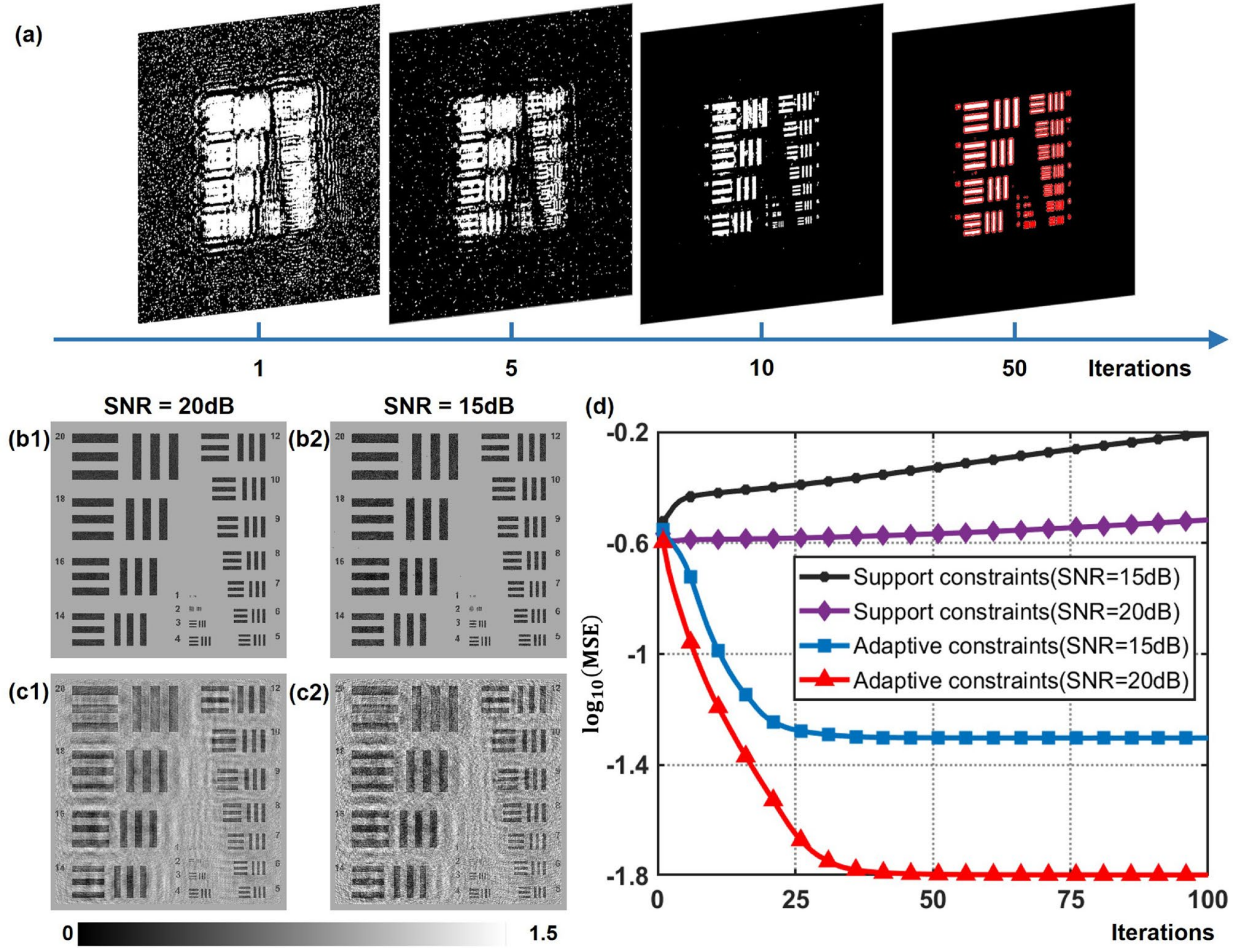
Reconstruction under white Gaussian noise with the SNR of 15 dB and 20 dB. (**a**) Evolutions of adaptive constraints under the case of a simulated noisy hologram with the SNR of 15 dB. (**b1**,**b2**) Reconstruction with adaptive constraints after 100 iterations under the condition of SNR = 20 dB and SNR = 15 dB respectively. (**c1**,**c2**) Reconstruction with support constraints after 100 iterations under the condition of SNR = 20 dB and SNR = 15 dB respectively. (**d**) Convergence behavior of support constraints and adaptive constraints with SNR = 20 dB and SNR = 15 dB.

Furthermore, single-shot phase retrieval inherits ill-posedness, which makes it susceptible to measurement noise. In order to evaluate the noise immunity of adaptive constraints implemented in the iterative phase retrieval, the simulated hologram is added white Gaussian noise with different signal to noise ratio (SNR). Evolutions of adaptive constraints when white Gaussian noise with the SNR of 15 dB is added to the simulated hologram are described in Fig. 5a. It is noteworthy that adaptive constraints updated automatically at each iteration contribute to sketching the contours of the object and filtering the reconstruction immersed in noise. Support constraints designed according to the object shape help to reduce noise outside the known boundary, but noise superimposed on the object within the support region still degrades the reconstruction quality, as seen in Fig. 5c1,c2. As depicted in Fig. 5b1,b2, reconstructions by adaptive constraints achieve a better recovery performance than using support constraints. To quantify the convergence behavior under noisy conditions, plot of logarithm MSE against iterations is performed in Fig. 5d. The reconstruction has a lower MSE with the increase of SNR. It can be observed that the MSE enlarges with the increasing of iterations by means of support constraints. That is because the noise within the support domain cannot be filtered out and accumulates during the iteration process, resulting in an increased reconstruction error. In contrast, adaptive constraints ensure convergence under moderate-noise conditions, having a better noise tolerance. Thus, using adaptive constraints is profitable to enhance the reconstruction quality and improve convergence behavior under noisy conditions.

## Experimental reconstruction

**Figure 6:**
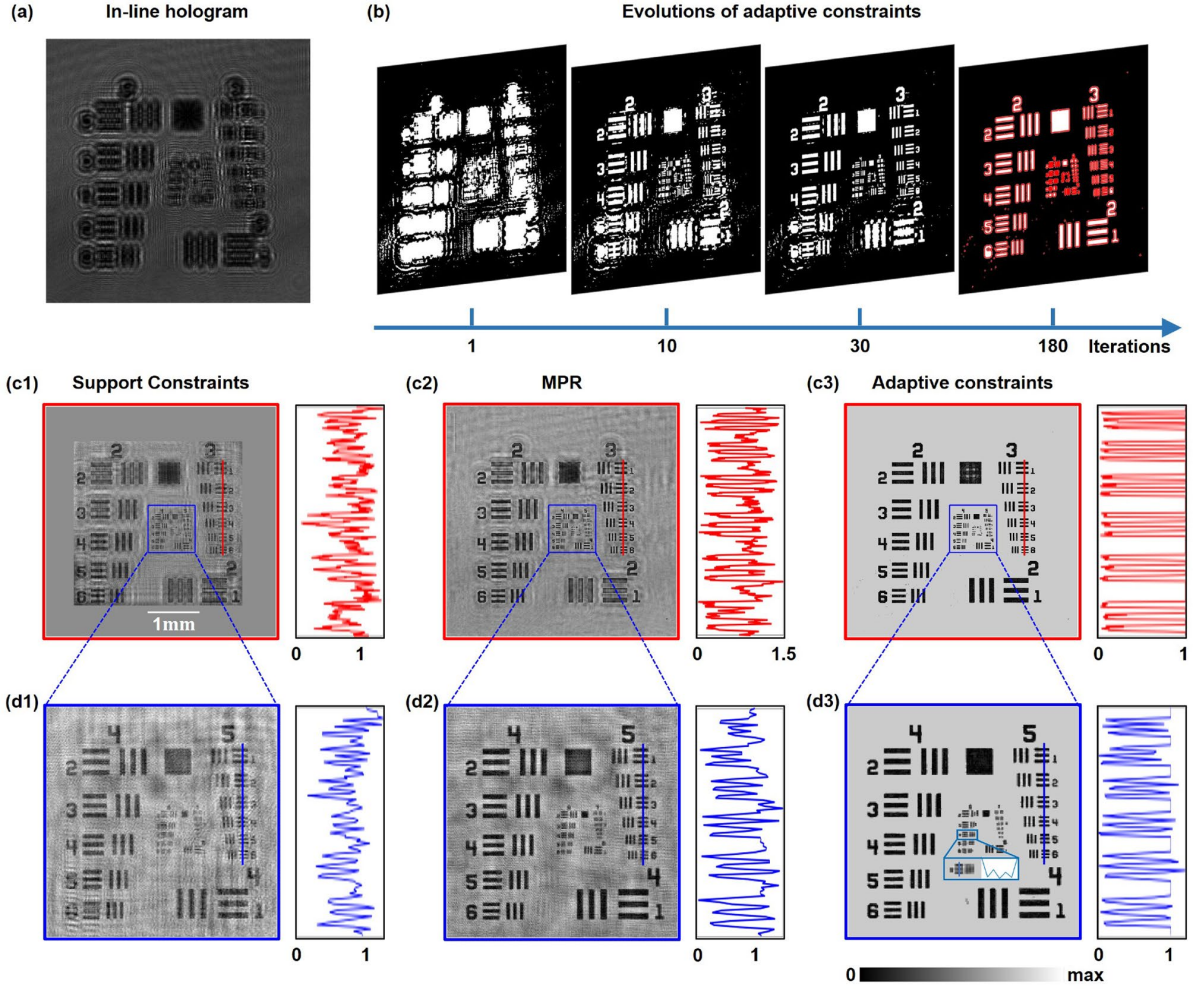
Experimental reconstruction of an amplitude resolution test target. (**a**) The captured in-line hologram. (**b**) Evolutions of adaptive constraints against iterations. (**c1**–**c3**) The retrieved amplitude by applying support constraints, MPR and adaptive constraints respectively after 200 iterations. Amplitude values of Group 3, Elements 1–6 are plotted on the right. (**d1**–**d3**) Reconstruction of the corresponding boxed areas in (**c1**–**c3**). Amplitude values of Group 5, Elements 1–6 are plotted on the right. The inset in (**d3**) represents the best structures resolved by adaptive constraints.

Next, we go further to experimentally confirm the improved reconstruction performance of our proposed method by imaging an amplitude resolution test target. By methods of nano processing, the positive 1951 USAF target (Thorlabs, Inc.) is fabricated by etching lines onto a glass plate. The raw in-line hologram in Fig. 6a is measured by the CMOS sensor at the distance of 4.14 cm from the object. For MPR method, the distance interval between three hologram planes is 0.5 mm. With the increasing of iterations, evolutions of adaptive constraints in Fig. 6b illustrate that introducing morphological operations helps to probe the structural features of the object to adaptively form a tight and sharp object support. Comparisons of the amplitude reconstruction by applying support constraints, MPR and adaptive constraints are performed in Fig. 6c1–d3. The retrieved amplitude by support constraints and MPR is severely obscured due to the existence of high reconstruction noise. By contrast, adaptive constraints can accomplish higher-quality reconstruction without artifacts overlapped, exhibiting better resolution with line width ranging from 8.77 to 15.63 $\upmu$m (Group 5, Elements 1–6). Also, Group 6, Element 3 of USAF 1951 with linewidth 6.2 $\upmu$m is the finest resolvable feature, which can be found in Fig. 6d3. It can be elucidated that measurement noise and the twin-image artifact which is interpreted as noise can both be removed by morphological operations.

**Figure 7:**
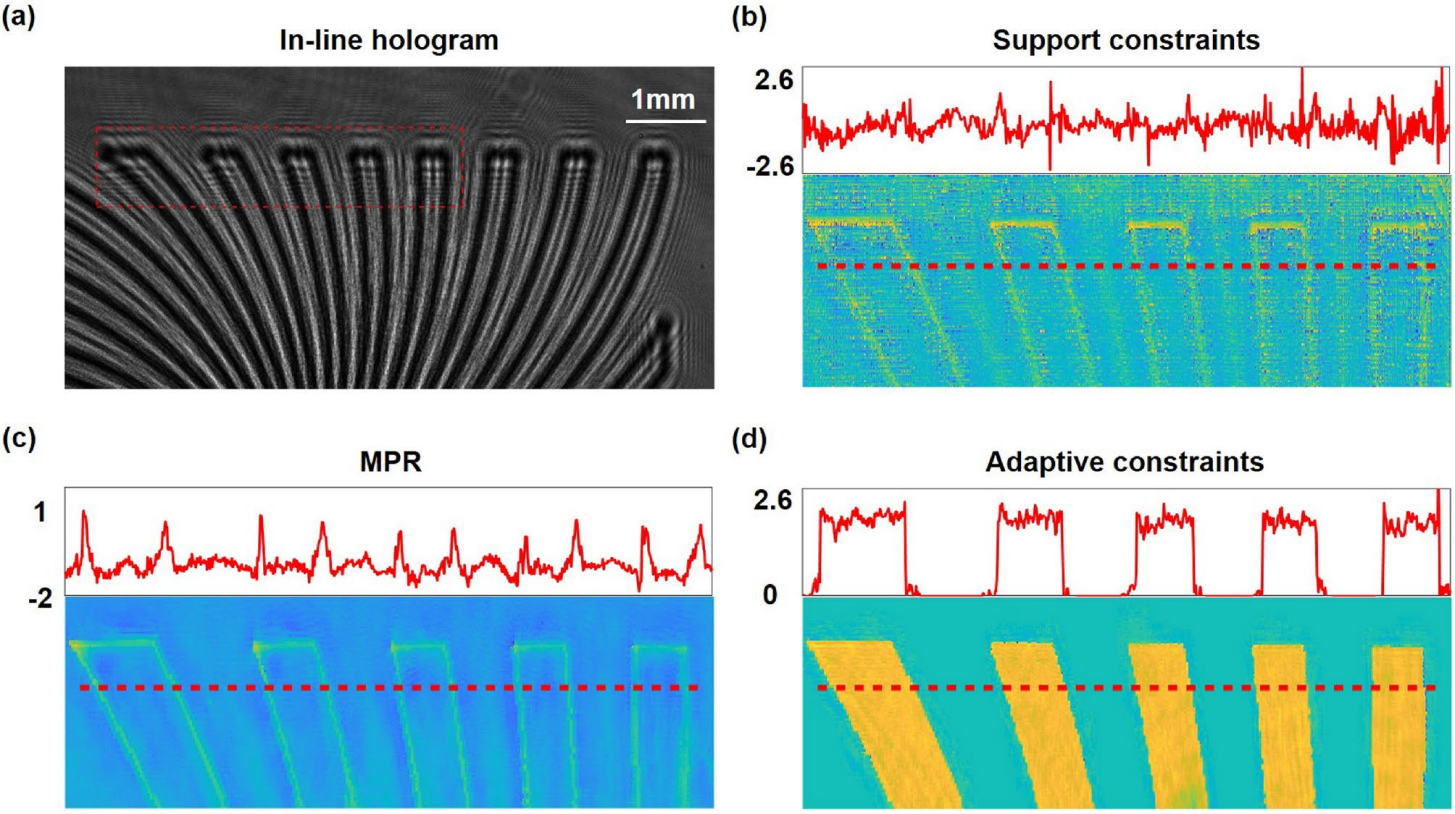
Experimental reconstruction of a phase plate. (**a**) The measured in-line hologram. (**b**–**d**) The surface profiles and the cross-sectional profiles of the retrieved phase corresponding to the boxed area by employing support constraints, MPR and adaptive constraints respectively after 500 iterations. The cross-sectional profiles show phase values along the red dotted lines in (**b**–**d**).

In addition, we imaged a phase plate to demonstrate the quantitative phase imaging ability of incorporating adaptive constraints. The phase plate is fabricated by etching multiple binary patterns onto a quartz glass plate. The raw in-line hologram in Fig. 7a is captured at the distance of 4.31 cm from the object. As observed in Fig.7b–d which present the surface profiles and the cross-sectional profiles of the retrieved phase by different approaches, the reconstruction quality by support constraints and MPR method is degraded attributing to the wrap-around artifact. Whereas enforcing adaptive constraints on the object domain is profit for eliminating twin-image artifacts from the retrieved phase distribution effectively. Besides, the low-frequency phase can hardly transfer into the intensity at the sensor plane because the frequency response of the weak phase transfer function declines to zero^60^, which poses challenges for recovering the low-frequency phase based on the in-line holographic system. It is noticed that iteratively updating adaptive constraints at the object plane seems to address this problem, which has a efficient reconstruction of the phase object. The experimental reconstruction confirms the improvement of reconstruction performance and the effectiveness in removing artifacts by exploiting adaptive constraints.

**Figure 8:**
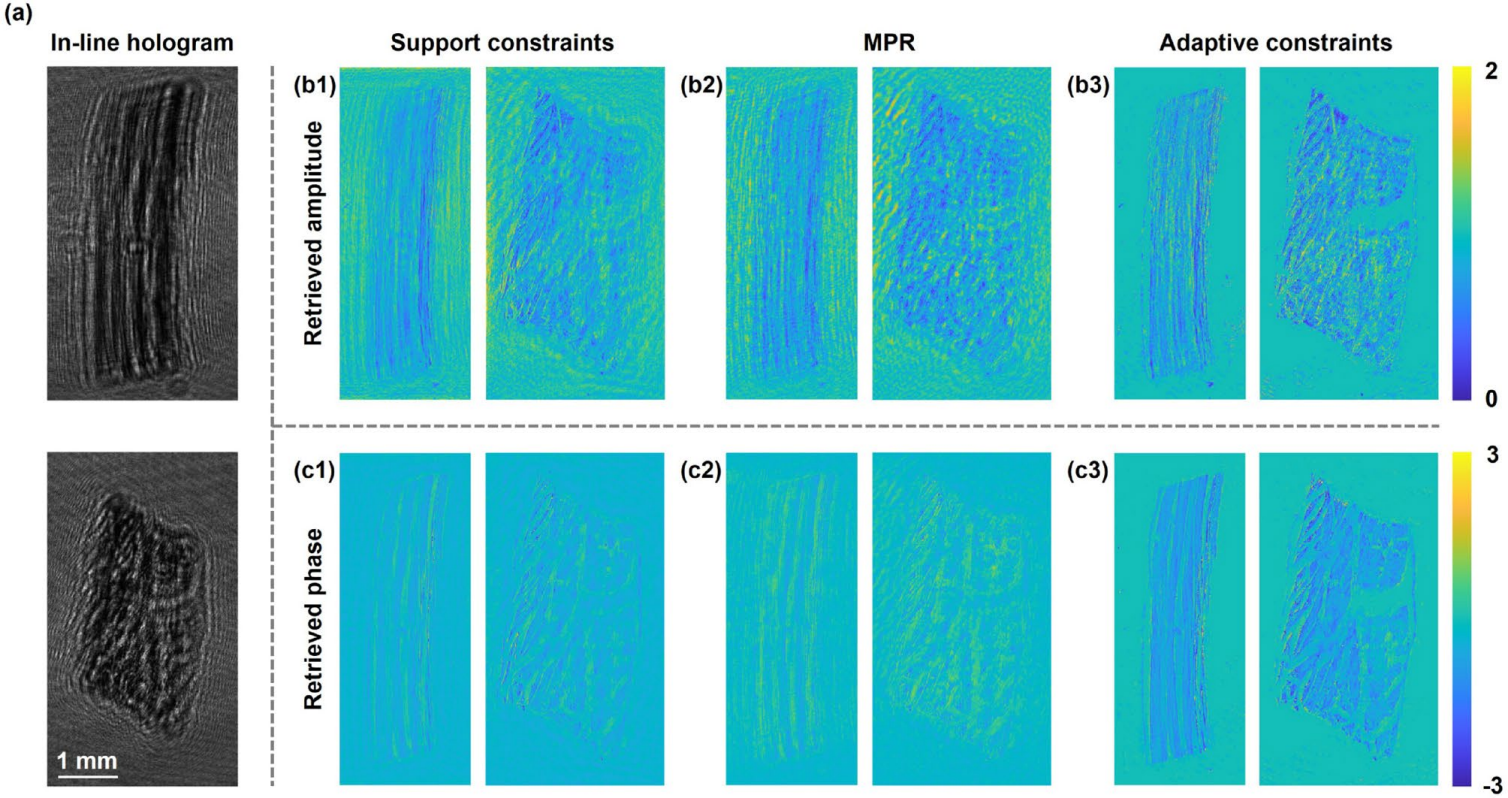
Experimental reconstruction of skeletal muscle cells. (**a**) The measured in-line hologram. (**b1**–**b3**) The retrieved amplitude by using support constraints, MPR and adaptive constraints respectively after 45 iterations. (**c1**–**c3**) The retrieved phase by using support constraints, MPR and adaptive constraints respectively after 45 iterations.

Furthermore, the skeletal muscle cells sandwiched between glass plates are selected to demonstrate the capacity of our proposed method to reconstruct complex-valued objects. Figure 8a shows the measured in-line holograms. Notice that the retrieved amplitude in Fig. 8b1–b3 and the retrieved phase in Fig. 8c1–c3 by methods of adaptive constraints present a better reconstruction than using support constraints and MPR. Using adaptive constraints can remove twin-image artifacts effectively and reveal the cell morphology clearly. For cell samples, too little light scattering from the specimen commonly results in a poor image contrast, which is difficult to bring out a distinguishable structure from an overwhelming incident light background^8^. The reconstructions performed in Fig. 8b3,c3 suggest that imposing adaptive constraints to confine the object support can significantly improve the image contrast and resolution.

## Conclusion

In summary, we propose an advanced iterative phase retrieval framework for single-shot in-line holographic imaging that incorporates adaptive constraints, which achieves high-fidelity reconstruction and optimizes convergence performance. Different from previous strategies for generating the object support, adaptive constraints by introducing morphological operations enable the object support to be automatically and accurately updated at each iteration. The capability of morphological filtering technique to extract the object structure while eliminating the irrelevant part provides a more appropriate and efficient estimation of support. Reconstruction of complex-valued objects by employing adaptive constraints is investigated and the immunity to noise is demonstrated. Compared with applying support constraints and MPR method, adaptive constraints are more effective in eliminating twin-image artifacts and speeding up convergence. Additionally, the improved reconstruction performance of this approach is experimentally confirmed by imaging an amplitude resolution target, a phase plate and skeletal muscle cells. Such flexible and versatile framework may better facilitate applications in biomedicine, X-ray coherent diffractive imaging and wavefront sensing.

## Data Availability

Data underlying the results presented in this paper are available in https://github.com/THUHoloLab/Adaptive-Constraints.
